# Early blockade of joint inflammation with a fatty acid amide hydrolase inhibitor decreases end-stage osteoarthritis pain and peripheral neuropathy in mice

**DOI:** 10.1186/s13075-017-1313-1

**Published:** 2017-05-25

**Authors:** Jason J. McDougall, Milind M. Muley, Holly T. Philpott, Allison Reid, Eugene Krustev

**Affiliations:** 1 Department of Pharmacology, Dalhousie University, 5850 College Street, Halifax, NS B3H 4R2 Canada; 2 Department of Anaesthesia, Pain Management & Perioperative Medicine, Dalhousie University, 5850 College Street, Halifax, NS B3H 4R2 Canada

**Keywords:** Endocannabinoids, Fatty acid amide hydrolase, Inflammation, Neuropathy, Osteoarthritis, Pain

## Abstract

**Background:**

The endocannabinoid system has been shown to reduce inflammatory flares and pain in rodent models of arthritis. A limitation of endocannabinoids is that they are rapidly denatured by hydrolysing enzymes such as fatty acid amide hydrolase (FAAH) which renders them physiologically inert. Osteoarthritis (OA) is primarily a degenerative joint disease; however, it can incorporate mild inflammation and peripheral neuropathy. The aim of this study was to determine whether early blockade of FAAH bioactivity could reduce OA-associated inflammation and joint neuropathy. The ability of this treatment to prevent end-stage OA pain development was also tested.

**Methods:**

Physiological saline or sodium monoiodoacetate (MIA; 0.3 mg) was injected into the right knee of male C57Bl/6 mice (20–42 g) and joint inflammation (oedema, blood flow and leukocyte trafficking) was measured over 14 days. Joint inflammation was also measured in a separate cohort of animals treated on day 1 with either saline or the FAAH inhibitor URB597 (0.03–0.3 mg/kg topical onto the knee joint). In other experiments, von Frey hair tactile sensitivity was determined on days 1 and 14 in MIA-injected mice treated prophylactically with URB597 (0.3 mg/kg s.c. over the knee joint on days 0–3). Saphenous nerve myelination was also assessed in these animals on day 14 by G-ratio analysis.

**Results:**

Intra-articular injection of MIA caused an increase in joint oedema (*P* < 0.0001), blood flow (*P* < 0.05), leukocyte rolling (*P* < 0.05) and adherence (*P* < 0.001) on day 1 after treatment which subsequently resolved over later time points. This acute inflammatory response was ameliorated by local URB597 treatment. Prophylactic local administration of URB597 prevented MIA-induced saphenous nerve demyelination, and chronic joint pain was also attenuated.

**Conclusions:**

These data indicate that local inhibition of FAAH in MIA-injected knees can reduce acute inflammatory changes associated with the model. Prophylactic treatment of OA mice with the endocannabinoid hydrolysis inhibitor URB597 was also shown to be neuroprotective and prevented the development of joint pain at later time points.

## Background

Chronic joint pain is a multifaceted process involving a complex interaction between joint neuropathophysiology and psychosocial influences. Patient-reported pain does not match the radiographic severity of joint disease [1] while objective pre-clinical studies have confirmed that nociceptor activity does not correlate with joint damage scores [2, 3]. This disconnect between joint destruction and symptom development makes pain management a complicated proposition for arthritis patients. It is currently thought that the pain associated with osteoarthritis (OA) differs between patients with varying degrees of nociceptive, inflammatory and neuropathic qualities [4]. Despite this mixed pain phenotype in OA, the first-line drug therapies used to treat joint symptoms are the non-steroidal anti-inflammatory drugs (NSAIDs). In general, OA joint pain responds better to NSAIDs than to paracetamol which has no anti-inflammatory properties [5]. As the disease progresses, however, the efficacy of NSAIDs declines and the need for higher doses of drugs exposes the patient to a greater risk of cardiovascular and gastrointestinal complications. Thus, safer approaches to ameliorate any inflammatory component of OA are required for long-term treatments.

Examination of joints harvested from OA patients and animal models reveals that the nerves innervating these joints have an abnormal morphology consistent with a peripheral neuropathy [6, 7]. Dorsal root ganglia (DRG) isolated from OA animals express the nerve injury marker activation transcription factor-3 (ATF-3), suggesting that a portion of the pain described in these animal could be neuropathic in nature [8, 9]. The biochemical pathways involved in joint neuropathy are obscure, although it has been found recently that the lipid mediator lysophosphatidic acid is a promising candidate [10]. This molecule, along with other mediators such as substance P, calcitonin gene-related peptide and tumour necrosis factor alpha, are upregulated during the inflammatory phase of the monoiodoacetate (MIA) model of OA [11]. In addition to causing joint pain and inflammation, these molecules are also capable of damaging neurones [12, 13]. Therefore, we hypothesize that acute inflammatory events in the MIA model will drive joint peripheral neuropathy at later time points leading to OA-related neuropathic pain.

The endocannabinoid system consists of endogenous molecules such as anandamide and 2-arachidonoylglycerol (2-AG) and two G protein-coupled receptors CB_1_ and CB_2_. Both receptors have been localized in knee synovium, where they are associated with sensory nerves [14, 15]. Pharmacological activation of CB_1_ receptors has been shown to reduce OA pain and increase joint blood flow [14, 16]. The role of CB_2_ receptors in OA pain modulation is less clear, due to the limitations of the available pharmacological tools. While the CB2 agonist GW405833 reduced mechanonociception in a normal joint, it actually had a sensitizing effect in OA joints causing an enhanced pain response [15]. Alternatively, intra-spinal injection of the CB2 agonist JWH133 reduced the central sensitization associated with OA [17], suggesting different sites of action for CB_2_ receptors in the control of OA pain. Endocannabinoids are known to accumulate in OA joints [18]; however, they are rapidly broken down by endogenous hydrolases such as fatty acid amide hydrolase (FAAH). Blockade of FAAH activity has been shown to reduce joint pain and inflammation in rodent models of arthritis, indicating that the endocannabinoid system may be an efficacious way of treating joint disease [19, 20].

The aim of the present investigation was to determine whether FAAH inhibition could reduce the incipient inflammatory phase associated with the onset of MIA-induced OA. Secondly, experiments were performed to test whether interfering with this initial inflammatory episode would improve pain perception at later times in the MIA model. Finally, a potential neuroprotective effect of the FAAH inhibitor was assessed to see whether such a treatment could alleviate MIA-induced neuropathy.

## Methods

### Animals

A total of 178 male C57BL/6 mice (20–42 g; Charles River, QC, Canada) were used in these experiments. Following arrival at the facility, all animals were allowed at least 1 week to acclimate to their new environment. Mice were housed in ventilated racks at 22 ± 2 °C on a 12:12-hr light:dark cycle (light on from 7:00 to 19:00). Cages were lined with woodchip bedding and animals were provided with environmental enrichment. Standard laboratory chow and water were provided ad libitum. All experimental protocols were approved by the Dalhousie University Committee on Laboratory Animals, which acts in accordance with the standards put forth by the Canadian Council for Animal Care.

### Sodium monoiodoacetate-induced osteoarthritis

Animals were deeply anaesthetized (2–4% isoflurane; 100% oxygen at 1 L/min). The right knee joint was shaved and swabbed with 100% ethanol, and then 10 μl of MIA (0.3 mg in saline) was injected into the synovial space. A separate cohort of control sham animals received an intra-articular injection of sterile saline. The knee was then manually extended and flexed for 30 s to disperse the solution throughout the joint. In separate groups of mice, inflammation was assessed on days 1, 3, 7, 10 and 14, while pain and saphenous nerve demyelination were assessed on day 14.

### Measurements of inflammation

#### Joint oedema

Joint oedema was assessed by measuring the distance between the medial and lateral femoral condyles using a digital micrometer (VWR, Friendswood, TX, USA). Measurements were taken immediately before intra-articular injection of MIA, and then again on the day of inflammation assessment. Triplicate measurements were carried out and the average joint diameter was calculated.

#### Synovial blood flow

Animals were deeply anaesthetized by an intraperitoneal injection of urethane (25% in saline; 0.25–0.4 ml). A longitudinal incision was made in the ventral skin of the neck to expose the trachea which was cannulated to permit unrestricted breathing. The left carotid artery and the left jugular vein were also cannulated with PE-10 tubing filled with heparinized saline (1 U/ml) to allow mean arterial pressure (MAP) monitoring and intravenous (i.v.) access, respectively. The articular microcirculation of the right knee was exposed by surgically removing a small ellipse of overlying skin and the hind limb was immobilized. Physiological buffer (37 °C) was immediately and continuously perfused over the exposed joint.

Laser speckle contrast analysis (LASCA) was used to measure synovial vascular perfusion using a PeriCam PSI System (Perimed Inc., Ardmore, PA, USA). Recordings of the exposed knee joint were taken at a working distance of 10 cm with a frame capture rate of 25 images per second. Using dedicated software (PIMSoft, Version 1.5.4.8078), images were averaged to generate one perfusion image per second and a 1-min LASCA recording was taken at each time point. At the end of the experiment, mice were euthanized and a dead scan of the knee was taken post mortem. This “biological zero” value was subtracted from all measurements to account for any optical noise in the tissue. Images were analysed offline where blood perfusion in a defined region of interest approximating the knee joint was calculated. Because resting MAP was different between animals, vascular conductance was calculated:

$$ \mathrm{Conductance}=\frac{\mathrm{blood}\;\mathrm{perfusion}}{\mathrm{mean}\;\mathrm{arterial}\;\mathrm{pressure}} $$

#### Intravital microscopy

Intravital microscopy (IVM) was used to assess leukocyte–endothelial interactions within the microcirculation of the knee joint, as described previously [21]. After surgical exposure of the joint, the synovial microcirculation was visualized under incident fluorescent light using a Leica DM2500 microscope with an HCX APO L 20× objective and an HC Plan 10× eyepiece with a final magnification of 200×. In-vivo leukocyte staining was achieved by intravenous administration of rhodamine 6G (R6G; 0.05%, 0.05 ml, saline) and fluorescent videos were captured for 1 min by a Leica DFC 3000 camera (Leica Microsystems Inc., ON, Canada). Straight, unbranched, post-capillary venules (15–50 μm in diameter) overlying the knee joint capsule were selected for visualization. Two measures of leukocyte–endothelial interactions were used to assess inflammation: the number of rolling leukocytes and the number of adherent leukocytes. Rolling leukocytes are defined here as R6G-stained cells travelling slower than the surrounding flow of blood in the vessel of interest. The rolling leukocyte measure was obtained by counting the number of rolling leukocytes per minute to pass an arbitrary line perpendicular to the vessel of interest. Adherent leukocytes were defined as R6G-stained cells that remained stationary for a minimum of 30 s. Total leukocyte adhesion was quantified by counting the number of adherent cells within a defined portion of the vessel (100 μm).

### Measurement of secondary allodynia

Von Frey hair algesiometry was used as a measure of referred nociception. Animals were placed in elevated Plexiglas chambers (30 cm long × 9 cm wide × 24 cm tall) on metal mesh flooring, which allows access to the plantar surface of the paws. After allowing the animal to acclimate until exploratory behaviour ceased, ipsilateral hind paw mechanosensitivity was assessed using a modification of the Dixon up-down method [22]. A von Frey hair of known bending force was applied perpendicular to the plantar surface of the hind paw (avoiding the toe pads) until it just bent, and then was held in place for 3 s. If there was a positive response (i.e. withdrawal, shake or lick of the hind paw), the next lower strength hair was applied; if there was no response, the next higher strength hair was applied up to a maximum cut-off level which corresponded to a 4-g bending force. After the first difference in response was observed, four further measurements were made. The 50% withdrawal threshold was determined using the formula:

$$ {10}^{\left[ Xf+ k\delta \right]}/10,000 $$

where *Xf* is the value (in log units) of the final von Frey hair used, *k* is the tabular value for the pattern of the last six positive/negative responses and δ is the mean difference (in log units) between stimuli.

### Myelination of saphenous neurones

A small section of saphenous nerve was excised post mortem just proximal to the ipsilateral knee joint and fixed for several days in 2.5% glutaraldehyde diluted with 0.1 M sodium cacodylate buffer. Samples were rinsed three times (10 min each) with 0.1 M sodium cacodylate buffer and fixed for 2 h in 1% osmium tetroxide. Nerves were then rinsed in distilled water, placed in 0.25% uranyl acetate at 4 °C overnight, dehydrated through a series of acetone washes (from 50 to 100%), then embedded in 100% epon araldite resin and placed in a 60 °C oven for 48 h to cure. Thin sections (100 nm) were cut and placed on mesh copper grids and stained as follows: 2% aqueous uranyl acetate (10 min), distilled water (2 × 5 min), lead citrate (4 min) and, finally, a distilled water rinse. Samples were viewed under a JEOL JEM 1230 transmission electron microscope at 80 kV. Representative images of saphenous nerve cross-sections were taken with a Hamamatsu ORCA-HR digital camera. For each nerve, one image containing a majority (50–100) of fibres was analysed. Axonal myelination was calculated by G-ratios using Image J software:

$$ \mathrm{G}\hbox{-} \mathrm{ratio} = \sqrt{\mathrm{internal}\ \mathrm{axonal}\ \mathrm{area}/\mathrm{entire}\ \mathrm{axonal}\ \mathrm{area}} $$

and values were averaged to give a mean G-ratio for each animal.

### Acute URB597 treatment for inflammation experiments

The effect of FAAH inhibition on MIA-induced inflammation was tested in day 1 MIA-injected mice. Following baseline IVM and LASCA measurements, URB597 (0.03–0.3 mg/kg) or vehicle (DMSO:cremophor:saline 1:1:8) was applied topically to the exposed mouse knee as a warm (37 °C) bolus (100 μl). Blood flow and leukocyte trafficking measurements were then carried out 20 min later.

### Prophylactic URB597 treatment for pain and nerve damage

#### Pain

Secondary allodynia was measured at days 1 and 14 in four cohorts of mice as follows: Group 1, intra-articular injection of saline (sham control); Group 2, intra-articular injection of MIA only (OA control); Group 3, MIA mice treated with vehicle once per day on days 0–3 (OA with prophylactic vehicle treatment); and Group 4, MIA mice treated with 0.3 mg/kg URB597 on days 0–3 (OA with prophylactic drug treatment). All vehicle and drug treatments for the pain experiments were administered locally (10 μl s.c.) around the knee joint.

#### Saphenous nerve myelination

Saphenous nerves were removed from three groups of mice consisting of day 14 saline sham control, day 14 MIA mice treated with local vehicle (10 μl s.c. around the joint) once per day on days 0–3 (OA with prophylactic vehicle treatment) and day 14 MIA mice treated with 0.3 mg/kg URB597 (10 μl s.c. around the joint) on days 0–3 (OA with prophylactic drug treatment).

### Drugs and reagents

URB597 (FAAH inhibitor; (3-(3-carbamoylphenyl)phenyl) *N*-cyclohexylcarbamate) was obtained from Cayman Chemicals (Ann Arbor, MI, USA). Rhodamine 6G, cremophor, dimethyl sulphoxide (DMSO), urethane and sodium monoiodoacetate were obtained from Sigma Aldrich (St. Louis, MO, USA).

URB597 (0.03, 0.3 and 3.0 mg/kg) was dissolved in vehicle (DMSO:cremophor:saline 1:1:8) on the day of use. MIA was dissolved in saline (0.3 mg/10 μl) on the day of use. Rhodamine 6G (0.05%) was dissolved in saline and stored in the dark at 4 °C. Physiological buffered saline (135 mM NaCl, 20 mM NaHCO_3_, 5 mM KCl, 1 mM MgSO_4_⋅7H_2_O, pH 7.4) was prepared in-house.

### Data presentation and analysis

The Gaussian distribution was assessed for each group using a Kolmogorov–Smirnov test. All data were normally distributed and were therefore analysed using parametric statistics (one-way or two-way ANOVA, Student’s *t* test). All data are presented as the mean ± the standard error of the mean (SEM) for *n* observations. *P* < 0.05 was considered statistically significant.

## Results

### Time course of MIA-induced inflammation

**Fig. 1:**
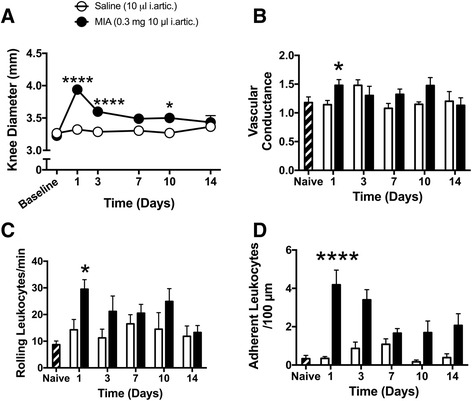
Early onset of joint inflammation in the MIA model of OA. **a** Knee joint diameter was significantly increased on days 1, 3 and 10 compared with saline control. **b** Joint blood flow increased on day 1, but subsequently was not significantly different from control joints. **c** Leukocyte rolling was augmented on day 1 and then returned to control levels thereafter. **d** Leukocyte adherence within synovial post-capillary venules was significantly increased on day 1, but gradually returned to control over the succeeding days. ****P < 0.0001, *P < 0.05 two-way ANOVA with Bonferroni post-hoc test; *n* = 4–28. Data presented as mean ± SEM. *i.artic.* intra-articular, *MIA* monoiodoacetate

When compared with saline-injected controls, intra-articular MIA (0.3 mg) significantly increased the knee joint diameter 1 day (*P* < 0.0001; *n* = 14–28; Fig. 1a) and 3 days (*P* < 0.0001; *n* = 6–11; Fig. 1a) after injection. Joint oedema subsequently returned towards control levels thereafter.

Intra-articular injection of MIA increased synovial vascular conductance on day 1 when compared with saline-injected controls (*P* < 0.05; *n* = 5–27; Fig. 1b). This hyperemic response was transient with blood flow returning to control levels from day 3 onwards.

Leukocyte rolling was significantly different between MIA and saline-injected knees on day 1 after injection (*P* < 0.05; *n* = 13–27; Fig. 1c). At later time points, however, there was no statistical difference between MIA and saline-treated mice (*P* > 0.05; *n* = 4–11). Leukocyte adherence was significantly higher at day 1 in MIA-injected knees compared with saline-injected controls (*P* < 0.0001; *n* = 15–27; Fig. 1d). Leukocyte adherence was not statistically different between control and arthritic joints at subsequent time points (*P* > 0.05; *n* = 4–11; Fig. 1d).

### Effect of URB597 on MIA-induced inflammation

**Fig. 2:**
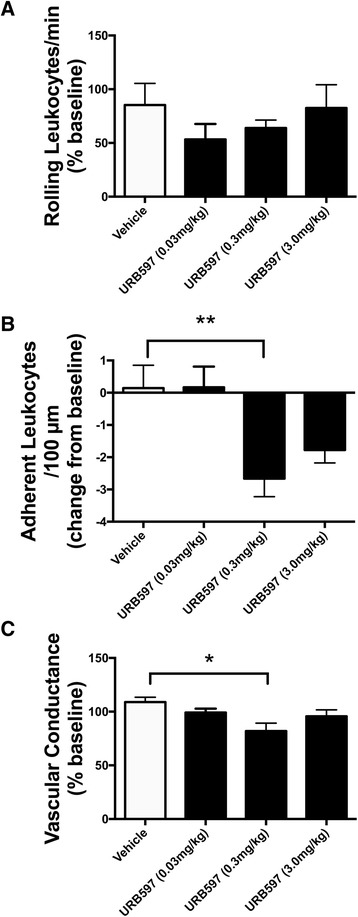
Local effect of URB597 on day 1 MIA-induced inflammation. **a** URB597 had no significant effect on leukocyte rolling when compared with vehicle. **b** URB597 (0.3 mg/kg) significantly decreased leukocyte adherence when compared with vehicle. **c** URB597 (0.3 mg/kg) significantly decreased knee joint blood flow when compared with vehicle. **P < 0.01, *P < 0.05 one-way ANOVA with Dunnett’s post-hoc test; *n* =5–8. Data presented as mean ± SEM

When compared with vehicle control, URB597 had no effect on leukocyte rolling at any of the doses tested (*P* > 0.05; *n* = 6–8; Fig. 2a). With leukocyte adherence, however, only the 0.3 mg/kg dose of URB597 caused a significant decrease (*P* < 0.01; *n* = 6–8; Fig. 2b), while the low and high doses of the drug were ineffective. Treatment of MIA-injected knees with 0.03 and 3.0 mg/kg URB597 had no effect on MIA-induced hyperaemia (*P* > 0.05; *n* = 6–8) whereas the 0.3 mg/kg dose of the drug significantly decreased articular blood flow (*P* < 0.05; *n* = 6–8; Fig. 2c).

### Effect of prophylactic URB597 on the chronic development of MIA-induced tactile allodynia

**Fig. 3:**
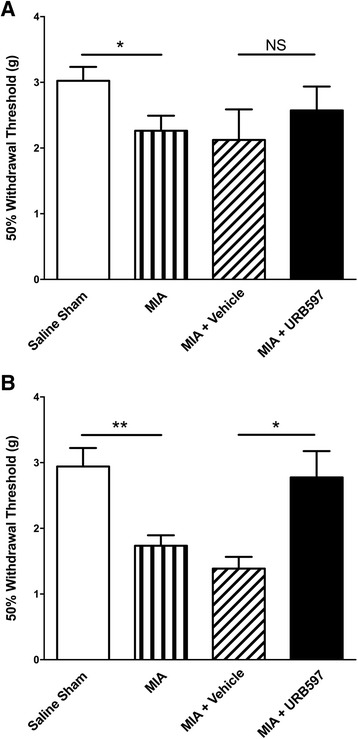
Effect of prophylactic URB597 treatment on the development of secondary allodynia after MIA injection. Intra-articular injection of MIA significantly reduced von Frey hair withdrawal threshold on day 1 (**a**) and day 14 (**b**) after injection compared with saline-injected knees. Treatment with URB597 (0.3 mg/kg) had no effect on day 1 secondary allodynia while prophylactic URB597 abolished chronic MIA-induced tactile hypersensitivity on day 14 compared with animals treated with a similar regimen of vehicle. **P < 0.01, *P < 0.05 unpaired two-tailed *t* test; *n* = 8–14. Data presented as mean ± SEM. *MIA* monoiodoacetate, *NS* not significant

Intra-articular injection of MIA produced secondary allodynia in the ipsilateral hind paw on days 1 and 14 after injection (*P* < 0.05; *n* = 8–15; Fig. 3a, b). On day 1, URB597 had no effect on von Frey hair mechanosensitivity (Fig. 3a). Prophylactic treatment of MIA-injected knees with URB597 on days 0–3, however, completely abolished the development of MIA-induced hypersensitivity during the chronic phase of the disease (*P* < 0.05; *n* = 8–10; Fig. 3b).

### Prophylactic effect of URB597 on MIA-induced saphenous nerve demyelination

**Fig. 4:**
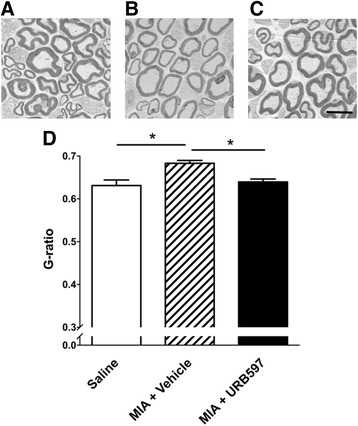
Prophylactic URB597 inhibits demyelination of joint nerves 14 days after MIA injection. Representative electron micrographs of axons found in saphenous nerves taken at day 14 from animals with a (**a**) saline-injected knee, (**b**) MIA-injected joint treated with vehicle (days 0–3) and (**c**) MIA-injected joint treated with URB597 (0.3 mg/kg, days 0–3). Myelin thickness is noticeably less in OA joint nerves and this demyelination is prevented by prophylactic URB597 treatment. **d** G-ratio calculations showing that MIA causes saphenous axonal demyelination which is prevented by URB597 treatment. *Scale bar* = 6 μm. *P < 0.05 one-way ANOVA with Tukey’s post-hoc test; *n* = 5–7. Data presented as mean ± SEM. *MIA* monoiodoacetate

Fourteen days after injection of intra-articular MIA, ipsilateral saphenous nerve axons showed a significant loss of myelin thickness, as evidenced by an increase in G-ratio (*P* < 0.05 compared with saline-injected controls; *n* = 5–7; Fig. 4). Repeated treatment of OA joints with URB597 during the acute phase of the model inhibited this end-stage demyelinating effect (*P* < 0.05; *n* = 6–7; Fig. 4).

## Discussion

Although OA is primarily a degenerative disease, many other pathophysiological features can contribute to the global symptoms of the disease. Acute inflammatory flares, for example, are associated with burning and aching sensations while chronic neuropathic symptoms tend to be stabbing and like electric shocks. We hypothesize that the acute inflammatory episodes found in OA drive peripheral neuropathy leading to a complex pain syndrome. The results presented here show that intra-articular injection of MIA produces an acute inflammatory response that resolves within 3 days. Thus, in the acute phase of this OA model there is a transient oedema response which is accompanied by an increase in synovial blood flow and leukocyte trafficking. These observations are similar to other reports which showed that intra-articular injection of MIA caused leukocyte infiltration and oedema which gradually resolved within days [23, 24]. This inflammatory response is thought to be mediated by transient receptor potential ankyrin-1 ion channel opening leading to the subsequent release of neuropeptides and joint neurogenic inflammation [24].

It has been reported previously that the FAAH inhibitor URB597 can reduce acute synovitis and joint neurogenic inflammation in mice [19, 25]. This anti-inflammatory effect of URB597 was reproducible in the current investigation which found that local administration of 0.3 mg/kg URB597 to MIA-injected knees decreased leukocyte adherence and hyperaemia. A lower and higher dose of URB597 failed to alter joint blood flow or leukocyte trafficking and this hormetic response is consistent with a previous finding in a model of acute inflammation [19]. The mechanism by which URB597 loses its efficacy at higher doses has yet to be investigated. One possibility is that the accumulation of endocannabinoids in the joint could trigger cyclooxygenase-2 activity which is known to lead to the production of pro-inflammatory prostamides [26], thereby offsetting any anti-inflammatory effects of FAAH inhibition. An alternative explanation is that high-dose URB597 or the resulting heightened endocannabinoid levels are having off-target effects. In joints, anandamide and the synthetic cannabinoid arachidonyl-2-chloroethylamide can sensitize articular transient receptor potential vanilloid-1 ion channels leading to nociception and hyperaemia [16, 27]. The net effect of these non-cannabinoid responses would be an attenuation of the anti-inflammatory and analgesic effects seen with the lower dose of URB597.

Local administration of URB597 to MIA-injected knees had no significant effect on the paw withdrawal threshold on day 1, suggesting that this particular FAAH inhibitor does not affect referred pain at the onset of this mouse model. Conversely, treatment of OA joints during the early inflammatory phase of the MIA model prevented the development of secondary allodynia at day 14. The pain generated at this later time point is, in part, due to the joint axonal damage that occurs in the MIA model. Thus, an acute intervention with URB597 during an inflammatory flare has the potential to protect the joint from chronic pain arising at later stages of the disease. Attenuating inflammation at the onset of the model could impede sensory bombardment within the dorsal horn of the spinal cord and avert central sensitization. Previous studies have found that central sensitization occurs in the late stages of the MIA model but not during early stages of the disease [28]. Future studies examining the effect of early URB597 on dorsal horn neuronal excitability in the chronic phase of MIA would help test this hypothesis. Although it is beyond the scope of this pain study, it would be interesting to see whether acute URB597 treatment could alter the course of end-stage joint destruction in OA, because a disease-modifying effect of the drug could also account for the observed prophylactic analgesia described here. While the present study focused on inflammation, pain and joint neuropathy, it would be interesting to see whether acute URB597 treatment could influence joint destruction; however, these histological experiments would be better carried out in a model of OA that more closely mimics the degenerative features of the human disease.

Peripheral neuropathy has been observed in the knees of OA patients [6] and in animal models of joint injury [7]. The present study discovered that MIA-induced OA caused demyelination of the ipsilateral saphenous nerve as evinced by an increase in G-ratio compared with sham control animals. Prophylactic treatment of MIA joints with locally administered URB597 during the inflammatory phase of the model prevented this loss in joint nerve myelination. This finding suggests that promoting endocannabinoids locally in the joint during the onset of OA development can protect the joint from neurodegeneration. Cannabinoids are involved in neurogenesis and are known to be neuroprotective in the nervous system [29, 30]. For example, cannabinoid receptor agonists can inhibit neuronal demyelination in a mouse model of multiple sclerosis by promoting interleukin-6 accumulation which is known to be neuroprotective [31, 32]. In a model of chemical neurotoxicity, the endocannabinoid hydrolysis inhibitor JZL184 reduced white matter lesions in the spinal cord of affected animals [33]. Interestingly in the same study, URB597 was unable to prevent oligodendrocyte death in vitro; however, the drug was not tested for its neuroprotective potential in vivo using a therapeutic regimen as described here.

Despite the promising data presented here and elsewhere, FAAH inhibition has yet to show a positive clinical effect for treating OA pain in patients. The irreversible FAAH inhibitor PF-04457845 was found to reduce pain in the MIA model [34], but failed to alleviate pain in a heterogeneous population of OA patients [35]. In these preclinical and clinical studies, however, the drug was given systemically, which increases the likelihood of imparting off-target effects in multiple organs. Conversely, the FAAH inhibitor presented here was administered locally around the joint, which would render a more targeted therapy and avoid non-articular side effects. A further limitation of the PF-04457845 clinical trial was that it was carried out on a mixed cohort of OA patients whose disease subtype was undefined. The data described in the present study suggest that FAAH inhibition may be more applicable to OA patients whose pain has a strong neuropathic component.

## Conclusion

Inhibition of the endocannabinoid hydrolysis enzyme FAAH results in a reduction in the acute inflammation that occurs at the onset of MIA-induced OA. By blocking this inflammatory response, URB597 reduces OA pain and prevents joint nerve damage during the chronic degenerative phase of the disease. These results suggest that early suppression of an inflammatory flare by articular endocannabinoids may be beneficial in attenuating the development of late-stage OA neuropathic pain.

## Abbreviations

2-AG: 2-Arachidonoylglycerol
ATF-3: Activated transcription factor-3
CB: Cannabinoid
DMSO: Dimethylsulphoxide
DRG: Dorsal root ganglia
FAAH: Fatty acid amide hydrolase
IVM: Intravital microscopy
LASCA: Laser speckle contrast analysis
MAP: Mean arterial pressure
MIA: Monoiodoacetate
OA: Osteoarthritis
